# Impact of acetylsalicylic acid on perioperative bleeding complications in deceased donor kidney transplantation

**DOI:** 10.1007/s00345-024-05426-y

**Published:** 2025-01-03

**Authors:** Frank Friedersdorff, Matthias Schulz, Sarah Weinberger, Scarlet Munayco Ramos, Bernhard Ralla, Lutz Liefeldt, Martin Kanne, Senem Sakar, Markus H. Lerchbaumer, Thorsten Schlomm, Isabel Lichy, Robert Peters, Jacob Schmidt

**Affiliations:** 1 Department of Urology, Charité – Universitaetsmedizin Berlin, Corporate Member of Freie Universitaet Berlin, Humboldt-Universitaet zu Berlin, Berlin Institute of Health, Charitéplatz 1, 10117 Berlin, Germany; 2 Department of Nephrology, Charité – Universitaetsmedizin Berlin, Corporate Member of Freie Universitaet Berlin, Humboldt-Universitaet zu Berlin, Berlin Institute of Health, Berlin, Germany; 3 Department of Urology, Evangelisches Krankenhaus Koenigin Elisabeth Herzberge, Berlin, Germany; 4 Department of Radiology, Charité – Universitaetsmedizin Berlin, corporate member of Freie Universitaet Berlin, Humboldt-Universitaet zu Berlin, Berlin Institute of Health, Berlin, Germany

**Keywords:** Kidney transplantation, Thrombosis, Bleeding, Anticoagulants, Acetyl salycilic acid

## Abstract

**Purpose:**

The objective of this study was to evaluate the perioperative outcomes and complications associated with the use of acetylsalicylic acid (ASA) in deceased donor kidney transplantation (KTX), with a particular focus on bleeding events.

**Methods:**

We retrospectively analyzed 157 kidney transplant recipients (KTRs) who underwent KTX at Charité Berlin, Department for Urology, between February 2014 and December 2017. Patients were divided into two groups: patients with ASA in their preoperative medication (Group A, *n* = 59) and patients without ASA use (Group B, *n* = 98). Data on demographic information, medical conditions, surgical details, and postoperative outcomes were analyzed. Complications were classified using the Clavien-Dindo classification. Statistical analyses included t-tests, chi-square tests, and multivariate logistic regression.

**Results:**

Group A had significantly older donors (59.7 ± 12.9 years vs. 52.0 ± 14.1 years, *p* < 0.001) and a higher incidence of coronary artery disease (42.4% vs. 3.1%, *p* = 0.001). There were no significant differences in perioperative hemoglobin loss and perioperative bleeding events between the groups, but a tendency towards higher rates of intraoperative bleeding (15.3% vs. 8.2%, *p* = 0.17) and postoperative transfusions (22% vs. 13.3%, *p* = 0.15) in Group A. Mortality was higher in Group A (18.6% vs. 4.1%, *p* = 0.003), with one death attributed to a cardiac event. Kaplan-Meier analysis revealed significantly inferior overall survival for Group A (*p* = 0.02), but no significant difference in graft survival (*p* = 0.18).

**Conclusion:**

ASA use is associated with a trend towards increased intraoperative bleeding and postoperative blood transfusion but does not significantly increase major postoperative bleeding complications. Careful perioperative monitoring of patients with ASA is recommended.

**Supplementary Information:**

The online version contains supplementary material available at 10.1007/s00345-024-05426-y.

## Introduction

Patients with end-stage renal disease (ESRD) commonly present with multiple comorbidities, including significant cardiovascular disease, which require the utilization of anticoagulation or antiplatelet therapy (AT) [1, 2]. Acetylsalicylic acid (ASA), is widely used for its antithrombotic properties to reduce the risk of cardiovascular events [3]. However, the administration of ASA during the perioperative period of kidney transplantation (KTX) presents a clinical dilemma: balancing the prevention of thrombotic events with the risk of perioperative bleeding, given the already increased risk of hemorrhagic events in patients with chronic kidney disease (CKD) [3–5]. Moreover, anticoagulation and AT cannot usually be discontinued in time for surgery, and most patients undergo surgery despite recent anticoagulation or AT [1].

Several studies have explored the impact of AT on KTX-outcomes, yielding inconclusive results. A meta-analysis by Cheungpasitporn et al. including 19,759 kidney transplant recipients demonstrated significant associations between ASA use and reduced risks of allograft failure and thrombosis, as well as major adverse cardiac events [6]. However, data on the risk of bleeding in kidney transplant recipients (KTRs) with ASA use were limited and inconclusive [6]. A retrospective analysis by Eng et al. revealed that ASA did not appear to increase transfusion rates but did result in an increased reoperation rate in their cohort [1]. Moreover, Benkö et al. reported a high bleeding complication rate of 31.5% in KTRs receiving AT with a revision rate of 50% when bleeding occurred [7]. This highlights the need for further investigation of ASA use and its impact on the potential benefits and risks in the perioperative setting of KTX.

In the present study, we retrospectively analyzed the perioperative outcomes of cadaveric donor KTX performed at a single high-volume transplant center, focusing on the incidence of bleeding complications in the context of AT.

## Materials and methods

### Patients

In the present retrospective study, we compared deceased donor KTRs with ASA in their preoperative medication regimen (Group A) versus no preoperative ASA medication (Group B). Overall 157 patients, who underwent KTX at Charité Hospital Berlin, Department for Urology, between February 2014 and December 2017 were included. Demographic information (gender, age, BMI, donor age), information on the patient’s medical history (primary kidney disease, pre-existing conditions, HLA mismatch, medication), surgical details (cold ischemia time (CIT), operative time of anastomosis, duration of surgery, implantation side), and postoperative outcomes were collected from the medical records. Complications were classified according the Clavien-Dindo classification (CDC) in the first 30 postoperative days [8]. For bleeding complications pre- and postoperative hemoglobin (Hb), Doppler ultrasound-detected hematoma and blood transfusions were analyzed. Intraoperative bleeding events were documented based on the surgeon’s description of bleeding events or an increased bleeding tendency in the operative report.

The use of ASA and anticoagulants including vitamin K antagonists (VKA), low molecular weight heparin (LMWH), heparin-dialysis on the day of surgery, and the use of marcumar antidotes before surgery, such as prothrombin complex concentrate and konakion were recorded. The standard dose of ASA as secondary prevention of cardiovascular disease was 100 mg/d and it was regularly continued during the clinical course. In some cases, the medication was paused based on a risk assessment that considered the specific indication for ASA as well as the bleeding risk during the clinical course. The standard procedure at our transplant center was to administer 250 IU/h unfractionated heparin as thrombosis prophylaxis, starting 6 h after the kidney transplantation.

### Statistical analysis

Statistical analysis of the data was performed using IBM SPSS Statistics 29 software. Student t-test was used for continuous variables and chi-square test for nominal variables. Kaplan-Meier analysis was done to compare survival data (log-rank test). Multivariate logistic regression was performed to identify independent risk factors for bleeding events controlled for potential confounders. A *p* value of < 0.05 was considered significant.

## Results

The cohort consisted of 157 patients, 59 of whom had ASA in their premedication (Group A) and 98 without ASA use as control group (Group B) (Table 1).

**Table 1:** Patient characteristics

Characteristics	Cohort (n = 157)	Group A (ASA, n = 59)	Group B (No ASA n = 98)	P-value
Gender				0.79
female	66 (42%)	35 (59.3%)	56 (57.1%)	
Male	91 (58%)	24 (40.7%)	42 (42.9%)	
Age (y)	54.9 ± 12.5	57.5 ± 12.2	53.5 ± 12.4	0.46
Donor Age (y)	54.9 ± 14.2	59.7 ± 12.9	52.0 ± 4.1	*<0.001
Eurotransplant Senior Program	25 (15.9%)	11 (18.6%)	14 (4.3%)	0.47
BMI (kg/m²)	26.1 ± 5.2	25.6 ± 4.5	26.4 ± 5.5	0.45
Primary Kidney Disease				*0.03
Other	14 (8.9%)	4 (6.8%)	10 (10.2%)	
Glomerulonephritis	56 (35.7%)	17 (28.8%)	39 (39.8%)	
Polycystic Kidney Disease	31 (19.7%)	10 (16.9%)	21 (21.4%)	
Systemic Diseases	12 (7.6%)	3 (5.1%)	9 (9.2%)	
Angiopathy	29 (18.5%)	14 (23.7%)	15 (15.3%)	
Kidney Infection	15 (9.6%)	11 (18.6%)	4 (4.1%)	
Pre-existing conditions				
Smoker				0.48
Unknown	47 (29.9%)	14 (23.7%)	33 (33.7%)	
Ex-Smoker	25 (15.9%)	9 (15.3%)	16 (16.3%)	
Non-Smoker	34 (21.7%)	13 (22%)	21 (21.4%)	
Smoker	51 (32.5%)	23 (39%)	28 (28.6%)	
Diabetes mellitus	20 (12.7%)	10 (16.9%)	10 (10.2%)	0.22
Coronary Artery Disease	28 (17.8%)	25 (42.4%)	3 (3.1%)	*0.001
Atrial Fibrillation	11 (7%)	6 (10.2%)	5 (5.1%)	0.23
Vein Thrombosis before	44 (28.0%)	20 (33.9%)	24 (24.5%)	0.20
Prior Abdominal Surgery	108 (68.8%)	45 (76.3%)	63 (64.3%)	0.12
Number of Surgeries	1.6 ± 1.7	1.8 ± 1.7	1.6 ± 1.7	0.44
Prior Kidney Transplantation	18 (11.5%)	5 (8.5%)	13 (13.3%)	0.36
1	14 (8.9%)	3 (5.1%)	11 (11.2%)	
2	4 (2.5%)	2 (3.4%)	2 (2%)	
HLA-Mismatch	2.27 ± 1.67	2.63 ± 1.60	2.04 ± 1.68	
Preoperative Anticoagulation	15 (9.6%)	5 (8.5%)	10 (10.2%)	0.72
VKA	9 (5.7%)	3 (5.1%)	6 (6.1%)	0.78
LMWH	3 (1.9%)	1 (1.7%)	2 (2.0%)	0.88
Heparin-Dialysis	3 (1.9%)	1 (1.7%)	2 (2.0%)	0.88
Preoperative VKA-Antidoting	5 (3.2%)	2 (3.4%)	3 (3.1%)	0.91

*Abbreviations* ASA, Acetyl Salicylic Acid; BMI, Body Mass Index; HLA, Human Leucocyte Antigen, VKA, vitamin K antagonists; LMWH, low molecular weight heparin

Values are shown as mean ± standard deviation or number (%); *p-value < 0.05 in students t-test or chi-square test

Patient characteristics including postoperative anticoagulation are shown in Table 1. There were no statistically significant differences between groups except for donor age (59.7 ± 12.9 years in the ASA group vs. 52.0 ± 4.1 years in the control group, *p* < 0.001), primary kidney disease (*p* = 0.03) and Coronary Artery Disease as a pre-condition (42.4% vs. 3.1%, *p* = 0.001).

**Fig. 1:**
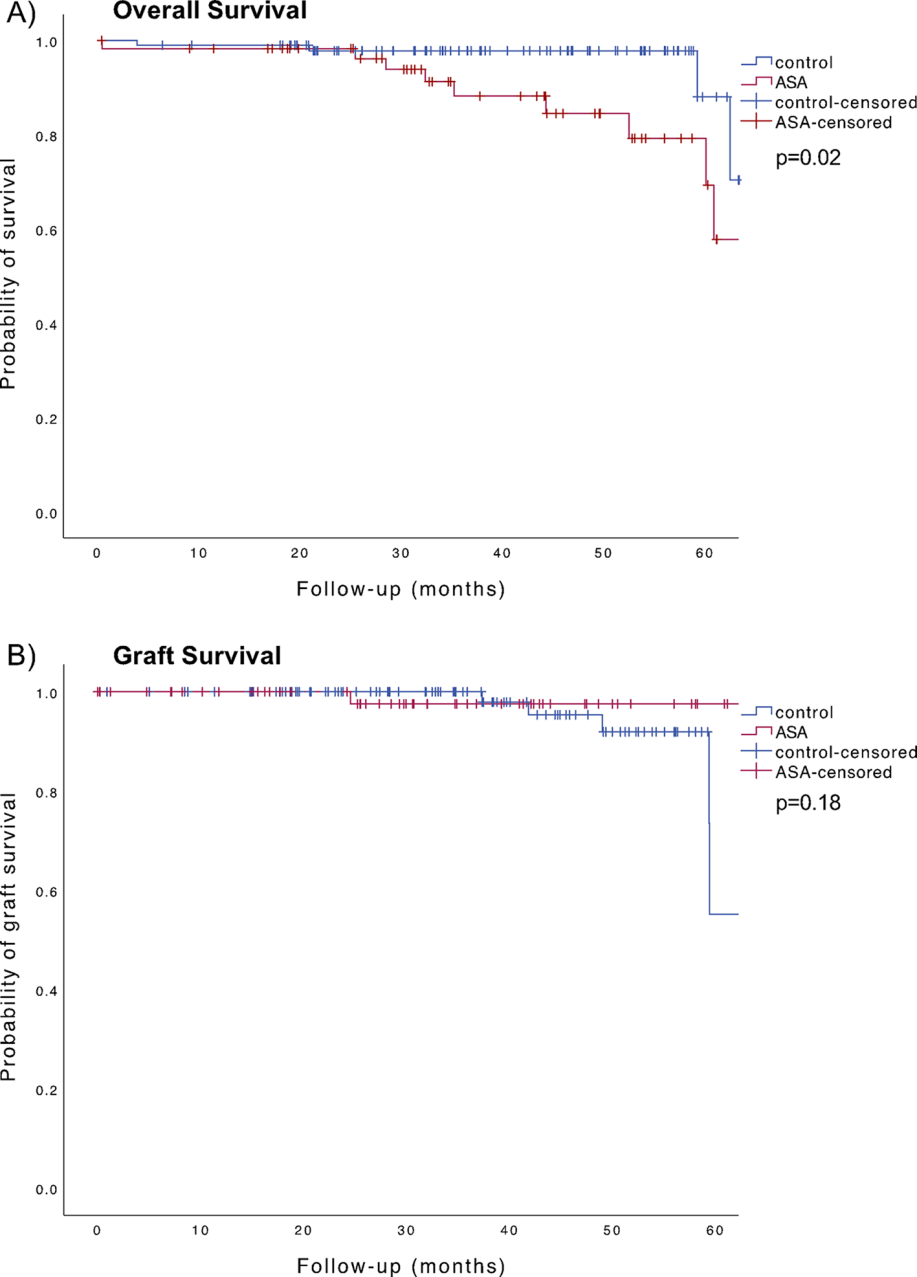
Kaplan Meier analysis of kidney transplant recipients with acetylsalicylic acid as part of their premedication (red) exhibited a significant decrease in overall survival (**A**) compared to the control group without ASA use (blue) (*p* = 0.02). No statistically significant differences were observed in graft survival (**B**) between both groups (*p* = 0.18)

Perioperative parameters and postoperative outcomes are shown in Table 2. Significant differences were classification of complications (Clavien Dindo, *p* = 0.01) and occurrence of death (18.6% vs. 4.1%, *p* = 0.003). The underlying type of major complication and reasons for revision are shown in Suppl. Table 1. The causes of death in the ASA group included infections in five cases, malignancies in four cases, a cardiac event, and intracerebral hemorrhage in one case. The cause of death was unknown in o case in each group. As shown in Fig. 1, survival analysis revealed a significantly inferior overall survival for Group A (*p* = 0.02), with a tendency towards improved graft survival in Group A, although not statistically significant (*p* = 0.18). As seen in Table 2, intraoperative bleeding was observed in 15.3% of patients in group A compared to 8.2% in group B (*p* = 0.17), although this difference was not statistically significant. Postoperative transfusion rates were higher in group A (22%) compared to group B (13.3%, *p* = 0.15). No significant differences were found in perioperative hemoglobin loss (0.93 ± 0.71 g/dL in group A vs. 1.06 ± 0.86 g/dL in group B, *p* = 0.31) or postoperative hematoma rates (28.8% vs. 26.5%, *p* = 0.75). At the time of discharge, ASA was discontinued in 15 cases (25.4%) within Group A. Multivariate logistic regression identified no independent risk factor for bleeding except female gender (regression coefficient B = 0.169, *p* = 0.032) (data not shown).

**Table 2:** Intraoperative parameters and postoperative outcomes

Characteristics	Cohort (*n* = 157)	Group A (ASA, *n* = 59)	Group B (No ASA *n* = 98)	*P*-value
Cold Ischemia Time (min)	743.6 ± 291.8	720.8 ± 281.5	757.7 ± 298.5	0.45
Time of Anastomosis (min)	46.8 ± 10.7	45.2 ± 9.6	47.7 ± 11.3	0.15
Duration of Surgery (min)	196.8 ± 42.1	193.0 ± 33.5	199.1 ± 46.5	0.38
Side of Implantation				0.34
left	63 (40.4%)	21 (35.6%)	42 (43.3%)	
right	93 (59.6%)	38 (64.4%)	55 (56.7%)	
Intraoperative Bleeding	17 (10.8%)	9 (15.3%)	8 (8.2%)	0.17
Intraoperative Transfusion	1 (0.6%)	0	1 (1%)	0.44
Hb-loss	1.0 ± 0.8	0.93 ± 0.71	1.06 ± 0.86	0.31
Bleeding p.o. (Hematoma)	43 (27.4%)	17 (28.8%)	26 (26.5%)	0.75
Transfusion p.o.	26 (16.6%)	13 (22%)	13 (13.3%)	0.15
Thrombosis p.o.	11 (7.0%)	3 (5.1%)	8 (8.2%)	0.46
Clavien Dindo Classification				*0.01
0	58 (36.9%)	15 (5.4%)	43 (43.9%)	
1	34 (21.7%)	14 (23.7%)	20 (20.4%)	
2	28 (17.8%)	16 (27.1%)	12 (12.2%)	
3	31 (19.7%)	9 (15.3%)	22 (22.4%)	
4	4 (2.5%)	3 (5.1%)	1 (1%)	
5	2 (1.3%)	2 (3.4%)	0	
Revision	27 (17.2%)	10 (16.9%)	17 (17.3%)	0.95
Delayed Graft Function	62 (39.5%)	22 (37.7%)	40 (40.8%)	0.66
Rejection	16 (10.2%)	6 (10.2%)	10 (10.2%)	0.99
Length of hospital stay (d)	17 ± 9	16 ± 8	17 ± 9	0.50
Graft loss	12 (7.6%)	4 (6.8%)	8 (8.2%)	0.75
Death	15 (9.6%)	11 (18.6%)	4 (4.1%)	*0.003
Follow up (m)	35.5 ± 16.1)	34.6 ± 16.4	36.0 ± 16.0	0.61

*Abbrevations* ASA, Acetyl Salicylic Acid; Hb, hemoglobin; p.o., postoperatively

Values are shown as mean ± standard deviation or number (%); *p-value < 0.05 in students t-test or chi-square test

## Discussion

Medications that affect blood coagulation, such as AT, are a critical component to consider in the clinical man-agement of patients undergoing KTX, requiring a delicate balance between minimizing thrombotic risk and mit-igating bleeding complications. This retrospective analysis aimed to provide a comprehensive evaluation of the impact of ASA monotherapy on perioperative bleeding in KTRs. Cadaveric donors for Group A were seven years older compared to Group B, reflecting the fact that a substantial number of patients in the ASA group were part of the ESP and therefore part of a more vulnerable KTX sub-cohort. Concordantly, Group A had a higher incidence of CAD, confirming the clinical rationale for ASA use in patients with cardiovascular comorbidities [9]. Key findings indicated that perioperative Hb-loss and intraoperative bleeding rates were not significantly different between both groups, and the incidence of postoperative bleeding indicated by sonographically detected hematomas did not differ (28.8% vs. 26.6%, *p* = 0.75). However, intraoperative bleeding (15.3% vs. 8.2%, *p* = 0.17) and the postoperative transfusion rate (22% vs. 13.3%, *p* = 0.15) were observed to be higher in the ASA group, although not statistically significant. This finding potentially reflects the cumulative effects of the intraoperative bleeding tendencies, which could lead to ongoing minor bleeding. The lack of significant differences in hemoglobin levels between the groups may be attributed to the limited sample size and the administration of erythrocyte concentrates in Group A, which likely contributed to aligning hemoglobin levels between the groups.

Consistent with our findings, Benkö et al. reported a prevalence of bleeding events in the same range, affecting 31.5% of their AT cohort [7]. In contrast, Eng et al. reported a higher transfusion rate of 28.8% in their cohort with preoperative ASA use, but, consistent with our results, did not show a significant difference compared with their control [1]. However, the ASA cohort of Eng et al. showed a 3-fold risk for reoperation, which is not reflected in our results with similar revision rates of 16.9% and 17.1%, respectively (*p* = 0.95) [1, 7]. Therefore, our results are consistent with those of Musetti et al., who showed an association of AT with a higher transfusion rate, but not with major bleeding events, and concluded that AT should not be considered a contraindication to KTX due to bleeding risk in itself [10]. Nevertheless, the need for close monitoring in cases of minor bleeding that may require transfusion is strongly emphasized in this patient group.

The vulnerability of our cohort is also highlighted by the results regarding the distribution of complications with fewer patients in the ASA group without complications (5.4% vs. 43%), and more patients with CDC Grade 4 and 5 complications (*p* = 0.01). It should be noted that, by indication for ASA, significantly more patients in Group A had CAD. In addition, angiopathies and infections as underlying ESRD were more common, and the predisposition to infection may be exacerbated by the need for immunosuppression. It is well known that cardiovascular disease and infections are major risk factors for severe complications and death after KTX [11, 12]. Accordingly, the severe complications and the significantly inferior OS of the ASA group must be seen primarily in the context of the underlying comorbidities and cannot be attributed to ASA use directly. This is validated by the results of our study, which indicate that nearly 50% of deaths in Group A were attributable to infections. It is noteworthy that only one death in Group A was related to a cardiac event, which appears low in comparison to the substantial number of CAD patients. Although a significant difference in OS was observed in our study, no significant difference in graft survival was found between the groups, with a tendency in favor of the ASA group in the Kaplan-Meier analysis. Therefore, our findings corrobate the results of the meta-analysis by Cheungpasitporn et al., who demonstrated that ASA use in KTRs is associated with a reduced risk of major cardiac events and appears to be protective against graft failure [6, 13].

Our analysis has several limitations. The retrospective design and single-center setting limit the generalizability. The study’s sample size, though moderate, may still be insufficient to detect small but clinically significant differences in rare outcomes. Moreover, the specific indications for ASA use were not determined in our study.

## Conclusion

While ASA use did not significantly increase the risk of major bleeding complications or transfusion, there were trends indicating a higher incidence of intraoperative bleeding and postoperative transfusions in the ASA group. Despite these trends, the potential benefits of ASA in reducing cardiac events are substantial, suggesting that its use can be particularly advantageous for graft and patient survival with cardiovascular risk. Nevertheless, vigilant monitoring of these patients is essential to mitigate the risks of bleeding complications.

## Electronic supplementary material

Below is the link to the electronic supplementary material.

Supplementary Material 1

## Data Availability

The data that support the findings of this study are available on request from the corresponding author.

## Abbreviations

AT: Antiplatelet therapy
ASA: Acetyl Salicylic Acid
BMI: Body Mass Index
CAD: Coronary Artery Disease
CDC: Clavien-Dindo classification
CIT: Cold ischemia time
CKD: Chronic kidney disease
ESP: Eurotransplant Senior Program
ESRD: End-stage renal disease
Hb: Hemoglobin
HLA: Human Leucocyte Antigen
KTR: Kidney transplant recipient
KTX: Kidney transplantation
LMWH: Low-molecular-weight heparins
OS: Overall survival
VKA: Vitamin K antagonists
WIT: Warm ischemia time
