# Supplementary material for: Impact of acetylsalicylic acid on perioperative bleeding complications in deceased donor kidney transplantation
Source: World J Urol. 2025 Jan 3;43(1):56. doi: 10.1007/s00345-024-05426-y (PMC11698840; doi:10.1007/s00345-024-05426-y)
Supplement: Supplementary file 1 — Supplementary Material 1 [file 345_2024_5426_MOESM1_ESM.docx]

**Supplementary material**

**Suppl. Table 1.** **Underlying major complication and reason for revision**; values are shown as number (%)

| **Characteristics** | **Cohort (n=157)** | **Group A**  **(ASA, n=59)** | **Group B**  **(No ASA n=98)** |
| --- | --- | --- | --- |
| **Major complication CDC ≥3** |  |  |  |
| Hydronephrosis with intervention | 2 (1.3%) | 2 (3.4%) | 0 |
| Renal arterial stenosis | 1 (0.6%) | 0 | 1 (1%) |
| Suprapubic urinary catheter | 1 (0.6%) | 0 | 1 (1%) |
| Major Bleeding | 8 (5%) | 3 (5.1%) | 5 (5.1%) |
| Fascial dehiscence | 12 (7.6%) | 4 (6.7%) | 8 (8.2%) |
| Cardiac event | 4 (2.5%) | 2 (3.4%) | 2 (2%) |
| Acute rejection | 2 (1.3%) | 0 | 2 (2%) |
| Thrombosis | 4 (2.5%) | 1 (1.7%) | 3 (3.1%) |
| Lymphocele | 1 (0.6%) | 0 | 1 (1%) |
| Anastomosis insufficiency | 1 (0.6%) | 1 (1.7%) | 0 |
| Ileus | 1 (0.6%) | 1 (1.7%) | 1 (1%) |
| Unknown | 1 (0.6%) | 0 | 1 (1%) |
| **Reason for revision** |  |  |  |
| Hydronephrosis with intervention | 1 (0.6%) | 1 (1.7%) | 0 |
| Renal arterial stenosis | 1 (0.6%) | 0 | 1 (1%) |
| Major Bleeding | 7 (4.5%) | 3 (5.1%) | 4 (4.1%) |
| Fascial dehiscence | 11 (7%) | 4 (6.7%) | 7 (7.1%) |
| Acute rejection | 1 (0.6%) | 0 | 1 (1%) |
| Thrombosis | 3 (1.9%) | 1 (1.7%) | 2 (2%) |
| Lymphocele | 1 (0.6%) | 0 | 1 (1%) |
| Anastomosis insufficiency | 1 (0.6%) | 1 (1.7%) | 0 |
| Ileus | 1 (0.6%) | 0 | 1 (1%) |

**Abbrevations:** ASA, Acetyl Salicylic Acid; CDC, Clavien Dindo Classification
